# Modeling and Validation of a LiOH Production Process by Bipolar Membrane Electrodialysis from Concentrated LiCl

**DOI:** 10.3390/membranes13020187

**Published:** 2023-02-02

**Authors:** Alonso González, Mario Grágeda, Svetlana Ushak

**Affiliations:** Departamento de Ingeniería Química y Procesos de Minerales, Center for Advanced Study of Lithium and Industrial Minerals (CELiMIN), Campus Coloso, Universidad de Antofagasta, Av. Universidad de Antofagasta, Antofagasta 02800, Chile

**Keywords:** lithium hydroxide production, bipolar membrane electrodialysis, high concentration LiCl solutions, modeling and process simulation

## Abstract

Electromembrane processes for LiOH production from lithium brines obtained from solar evaporation ponds in production processes of the Salar de Atacama are considered. In order to analyze high concentrations’ effect on ion exchange membranes, the use of concentrated LiCl aqueous solutions in a bipolar membrane electrodialysis process to produce LiOH solutions higher than 3.0% by mass is initially investigated. For this purpose, a mathematical model based on the Nernst–Planck equation is developed and validated, and a parametric study is simulated considering as input variables electrolyte concentrations, applied current density, stack design, process design and membrane characteristics. As a novelty, this mathematical model allows estimating LiOH production in a wide concentration range of LiCl, HCl and LiOH solutions and its effect on the process, providing data on final LiOH solution purity, current efficiency, specific electricity consumption and membrane performance. Among the main results, a concentration of 4.0% to 4.5% by LiOH mass is achieved, with a solution purity higher than 95% by mass and specific electrical energy consumption close to 4.0 kWh/kg. The work performed provides key information on process sensitivity to operating conditions and process design characteristics. These results serve as a guide in the application of this technology to lithium hydroxide production.

## 1. Introduction

In recent years, lithium hydroxide production has increased due to high projections in battery cathode manufacturing [1] because of high power density, longer life cycle and better lithium battery safety compared to other different chemistry batteries [2,3,4,5].

In industrial production, lithium hydroxide is obtained from lithium carbonate by reacting with calcium hydroxide according to the reaction ${\mathrm{Li}}_{2}{\mathrm{CO}}_{3}+\mathrm{Ca}{\left(\mathrm{OH}\right)}_{2}\to 2{\mathrm{LiOH}}_{\left(\mathrm{aq}\right)}+{\mathrm{CaCO}}_{3}{}_{\left(s\right)}$. As a product of this reaction, an aqueous solution is obtained that reaches a concentration close to 3% LiOH by mass, which is later sent to an evaporation and crystallization stage to obtain lithium hydroxide monohydrate. The initial lithium carbonate required for this reaction is obtained mainly from concentrated lithium brines 5.5–6.0% by mass from solar evaporation ponds [6]. Conventional LiOH production process from brine and intermediate Li_2_CO_3_ production requires several stages with different degrees of complexity and a significant amount of equipment. The latter involves energy costs, chemical reagents consumption and various solid and liquid waste generation [7].

Electrochemical processes with ion exchange membranes are a real alternative for value-added compound production, avoiding the use of chemical reagents. They can be coupled to non-conventional renewable energy systems, contributing to the adoption of an energy mix with a lower carbon footprint. Among them, the chlor-alkali process is an electromembrane technology currently used in NaOH production [8,9,10]. It uses a cation exchange membrane between two electrodes and, by electric current application, water reduction at the cathode is achieved to produce NaOH generating H_2_ gas through a semi-reaction: $2H_{2}O+2e^{-}\to H_{2}+2{\mathrm{OH}}^{-}$. In lithium recovery and LiOH production, Grageda et al. [11] have analyzed a membrane electrodialysis process to produce high-purity lithium hydroxide determining the effect of concentration, current density and temperature on process energy performance and product purity, reporting a specific electricity consumption of 7.25 kWh/kg LiOH. Similar work has been conducted by Ryabtsev et al. [12] in which they obtained an average 45 g/L LiOH concentration by membrane electrolysis from lithium carbonate treated with sulfuric acid taking advantage of Li_2_SO_4_ high solubility.

Bipolar membrane electrodialysis (BMED) [13,14,15,16], has been studied for desalination [17,18,19], sodium hydroxide production [20,21,22], acid production [23,24,25,26], acetates [27] and boron removal [28]. BMED main feature is the bipolar membrane's ability to generate H^+^ and OH^−^ protons by water dissociation reducing O_2_ and H_2_ gas generation and reducing electrodes associated costs [13,14,15,16]. In recent years, due to the increasing demand for lithium, studies of recovery from waste streams using conventional electrodialysis [29] and bipolar membrane electrodialysis recovering lithium as LiOH [30,31,32,33] have emerged. Among the main LiOH production works, Jiang et al. [34] obtained a LiOH solution with 95% purity and a specific electrical energy consumption of 6.66 kWh/kg from 0.18 M Li_2_CO_3_ aqueous solutions. On the other hand, Melnikov et al. [35], applied bipolar membranes electrodialysis at a pilot scale for obtaining LiOH from LiCl solution contaminated with organic solvents reaching a LiOH concentration of 0.3 M, a current efficiency of 60% and a specific energy consumption of 6.6 kWh/kg. Cho et al. [36] studied LiOH production from aqueous Li_2_SO_4_ solutions by simultaneously concentrating H_2_SO_4_. Recently, González et al. [37] applied bipolar membrane electrodialysis for LiOH production from concentrated lithium chloride solutions (greater than 14% by mass) achieving a LiOH solution of 4.35 mass % (approximately 1.9 M) with a 95.4% purity and a specific 9.45 kWh/kg electricity consumption. In the last years, Tian et al. [38] succeeded in obtaining LiOH concentrations of 3.57 M by experimenting with initial solutions of 70–130 g/L LiCl. On the other hand, Chen et al. [39], managed to concentrate LiOH up to 2.2 M from 1.5 M Li_2_SO_4_ solutions, reporting a specific electricity consumption of 7 kWh/kg and a LiOH solution purity close to 99.75%.

The Main BMED processes components are the bipolar membrane and the cation and anion exchange membranes, where ion transport in each is affected by membranes characteristics and their interaction with different operating conditions such as current density, concentration and electrolytes chemical composition, among others [13]. In the case of base production by BMED, OH^−^ ion leakage in cation exchange membranes has been reported to decrease current efficiency and therefore reduce process performance [40,41]. On the other hand, bipolar membrane performance is associated with the permselectivity of their anionic and cationic layers. Salt leakage can occur, limiting H^+^ protons and OH^−^ anions production efficiency [42,43].

BMED application for lithium hydroxide production from concentrated LiCl brines represents an implementation opportunity for a complementary green process for lithium compounds production due to its potential integration with solar photovoltaic energy [26]. To obtain LiOH solutions at concentrations higher than 3.0 mass %, it is necessary to overcome existing membrane limitations at high concentrations such as OH^−^ ion leakage in the cationic membrane and salt leakage in bipolar membranes [37]. Laboratory experimental study is useful for direct measurement of different parameters effects in the process such as electrolyte concentration and conductivity, electric current and voltage, among others. However, it can be expensive due to the chemical reagents' high consumption to prepare concentrated solutions in addition to the current high costs of commercial bipolar and monopolar membranes. Given demonstrated technical feasibility of LiOH production by BMED [37], it is necessary to develop tools to estimate process performance under different conditions.

Currently, few mathematical modeling studies of bipolar membrane electrodialysis processes have been covered as simulation tools [44,45,46,47], most of them being developed in recent years. Recently, the work of Culcasi et al. [47] stands out for developing a mathematical model of NaOH production according to different operating conditions and process scenarios at different scales. Regarding lithium transport models, Asadi et al. [48] presented a model of electrodialysis in a three-compartment cell applied to Li_2_SO_4_ solutions concentrated between 500 and 1000 mol/m^3^, simulating the transport of species through cation and anion exchange membranes. As a product, they obtain a LiOH solution concentrated between 813 and 908 mol/m^3^. On the other hand, with respect to the bipolar membrane, Panzo et al., [49] developed a specific two-dimensional steady-state model to describe the behavior of a bipolar membrane in the dissociation of water considering NaCl, HCl and NaOH solutions between 0.25 and 0.50 mol/L. Bipolar membrane properties such as porosity and tortuosity values were considered.

In order to evaluate BMED process performance at high concentrations and under various operating conditions, a BMED mathematical model for LiOH production from concentrated LiCl solutions was developed in this research. Compared to other mathematical models, the one proposed in this work differs in that it combines as input variables operating conditions and membrane characteristics with stack and process design conditions, with the objective of LiOH production from concentrated LiCl solutions (3600 to 9800 mol/m^3^). As far as the authors are concerned, this is the first specific mathematical model to simulate and estimate the performance of a LiOH production process at high concentrations. The developed model, based on the Nernst–Planck equation, is novel in that it takes into account migration, diffusion and counterion concentration effects on the membrane in contact with concentrated LiOH and LiCl solutions. It allowed estimating LiOH production, Li^+^ migration, OH^−^ generation and energy yield (current efficiency, Li^+^ transport number and specific electrical energy consumption), as a function of various operating parameters (concentration, current density, treated volume and stack design) and according to different membrane characteristics (fixed charge density, thickness and diffusion coefficients) for a wide concentration range of LiCl, LiOH and HCl solutions. The transport of species simultaneously on the cationic and bipolar membrane over a wide concentration range is simulated. In addition, by means of LiOH processes simulation by BMED, a parametric analysis is performed in order to determine operational conditions and membrane characteristics that permit a better guide for technology application in obtaining concentrated LiOH solutions with high purity.

## 2. Materials and Methods

### 2.1. Mathematical Model Development

**Figure 1:**
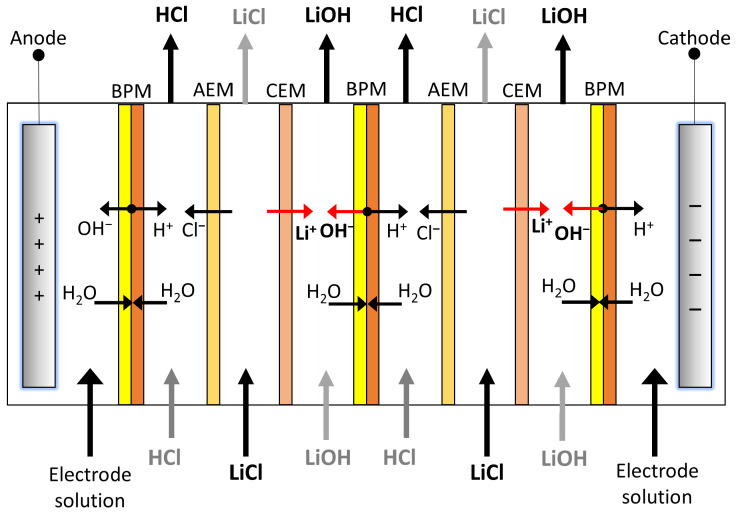
LiOH production scheme from LiCl by BMED with two compartments. BPM: Bipolar membrane, AEM: Anion Exchange membrane, CEM: Cation Exchange membrane.

Model development is based on lithium migration through the cation exchange membrane and OH^−^ ion generation in the bipolar membrane, which are the key components in LiOH production. Undesired phenomena such as OH^−^ leakage in cationic membranes and salt (Cl^−^) leakage in bipolar membranes are also simulated. In the process, an initial aqueous lithium chloride solution is used from which lithium is transported to the LiOH compartment and chloride ion to the HCl compartment, while H^+^ protons and OH^−^ ions are generated in the bipolar membrane (see Figure 1). In this way, the LiCl solution is diluted while LiOH and HCl solutions are concentrated. The basic stack unit is called a three-compartment cell and consists of a bipolar membrane (BPM), a LiOH compartment, a cation exchange membrane (CEM), a LiCl compartment, an anionic membrane (AEM) and an HCl compartment. In the present text, the “number of compartments” is used in reference to this basic process unit.

#### 2.1.1. Lithium Transport across the Cation Exchange Membrane

To determine lithium flux in cation exchange membranes in the mathematical model, the Nernst–Planck equation is used, along with several assumptions. First, it is considered that lithium transport depends mainly on phenomena occurring within cation exchange membrane boundaries, while diffusion boundary layer effects are neglected. Thus, lithium flux in the cation exchange membrane ($J_{Li}$) is given by the Nernst–Planck Equation (1), considering diffusion transport and migration. Similarly, the undesired flux of co-ions across this membrane is represented by hydroxide anion ($J_{OH}$) in Equation (2). Linear concentration profiles in membranes are considered, with ion transport being driven by concentration difference and electric potential difference $\left(\Delta \varphi_{cm}\right)\;$ on both membrane sides.

(1) $$J_{Li}=-D_{Li}^{cm}\frac{\Delta C_{Li}^{cm}}{\Delta x}-D_{Li}^{cm}\frac{z_{Li}C_{Li}^{cm}F}{RT}\frac{\Delta \varphi_{cm}}{\Delta x}$$

(2) $$J_{OH}=-D_{OH}^{cm}\frac{\Delta C_{OH}^{cm}}{\Delta x}-D_{OH}^{cm}\frac{z_{OH}C_{OH}^{cm}F}{RT}\frac{\Delta \varphi_{cm}}{\Delta x}$$

$D_{Li}^{cm}$ and $D_{OH}^{cm}$ represent the apparent diffusion coefficient in the cation exchange membrane for Li^+^ and OH^−^, respectively. Similarly, $C_{Li}^{cm}$ and $C_{OH}^{cm}$ are Li^+^ and OH^−^ concentrations in the membrane, Δx is the membrane thickness, z is the ion’s charge ($z_{Li}=1$, $z_{OH}=-1$), F is the Faraday’s constant (96,485 A∙s/mol), R is the ideal gas constant (8.314 J/mol/K), T is the absolute temperature and $\Delta \varphi_{cm}$ represents electric potential difference across the cation exchange membrane. Index “cm” indicates that term corresponds to cation exchange membrane.

Electric current density (i) can be expressed as a function of charge and ion flux through the cation exchange membrane according to Equation (3):

(3) $$i=F\left(\sum z_{i}J_{i}\right)$$

On the other hand, the neutrality principle relates to counterions concentration ($C_{cou}^{cm}$), co-ions ($C_{co}^{cm}$) and fixed charge density of the cation exchange membrane ($C_{fix}$) according to Equation (4).

(4) $$C_{cou}^{cm}=C_{co}^{cm}+C_{fix}$$

Due to the electroneutrality principle in the membrane, counterions concentration is proportional to co-ions concentration. Therefore, concentration differences in membrane boundaries are equivalent for both counterions and co-ions, i.e., $\Delta C_{Li}^{cm}=\Delta C_{OH}^{cm}$. This is defined by Equation (5):

(5) $$\Delta C_{Li}^{cm}=C_{Li}^{LiOH,cms}-C_{Li}^{LiCl,cms}$$

where $C_{Li}^{LiOH,cms}$ and $C_{Li}^{LiCl,cms}$ represent Li^+^ concentration on the cation exchange membrane faces in contact with LiOH and LiCl solutions, respectively.

From Equations (1)–(5) we obtain an expression for the electric potential difference in the cation exchange membrane ($\Delta \varphi_{cm}$), presented in Equation 6. This allows us to determine the value of $\Delta \varphi_{cm}$ as a function of applied current density $\left(i\right)$.

(6) $$\frac{\Delta \varphi_{cm}}{\Delta x}=-\frac{RT}{F\left(\left(D_{Li}^{cm}+D_{OH}^{cm}\right)C_{Li}^{cm}-D_{OH}^{cm}C_{fix}\right)}\left[\frac{i}{F}-\left(D_{OH}^{cm}-D_{Li}^{cm}\right)\frac{\Delta C_{Li}^{cm}}{\Delta x}\right]$$

Counterions concentration in the membrane depends on fixed charge density, concentration and ionic activity of species present in electrolytes. For practical purposes, in this mathematical model, the Donnan exclusion is used, assuming the ion activity effect in the membrane to be negligible according to Equation (7). This may give some deviation from real behavior. However, it is useful for parametric analysis of process and production estimates.

(7) $$C_{cou}^{cm}=\frac{C_{fix}}{2}+\sqrt{\frac{C_{fix}^{2}}{4}+C_{s}^{2}}$$

$C_{cou}^{cm}$ is counterions concentration on the cationic membrane, $C_{fix}$ is fixed charge density on the membrane and $C_{s}$ is aqueous solutions concentration in contact with the membrane. Equation (7) is used to determine lithium concentration as counterion on membrane faces in contact with LiOH and LiCl solutions ($C_{Li}^{LiOH,cms}\;and\;C_{Li}^{LiCl,cms}$), respectively. While counterions concentration on the cation exchange membrane ($C_{Li}^{cm}$) is considered an average calculated concentration for membrane faces due to both solutions.

Lithium transport across the cation exchange membrane causes ion concentration in LiOH solution and dilution in LiCl solution. Li^+^ concentration variation in LiCl solution $\left(C_{Li}^{LiCl}\right)$ and in LiOH solution can be related by Equation (8), assuming no lithium losses in transport across the cation exchange membrane.

(8) $$C_{Li}^{LiCl}=C_{Li,0}^{LiCl}+\frac{V_{LiOH}}{V_{LiCl}}\left(C_{Li,0}^{LiOH}-C_{Li}^{LiOH}\right)$$

where $C_{Li,0}^{LiCl}$ and $C_{Li,0}^{LiOH}$ are the initial concentrations of lithium in LiCl and LiOH solutions, while $V_{LiCl}$ and $V_{LiOH}$ are their corresponding volumes.

By combining and developing Equations (1) and (6)–(8), an expression for lithium flux through the cation exchange membrane is obtained (Equation (9)). This equation allows for calculating the lithium-ion flux of moles ($J_{Li}$) according to applied current density (i) and LiOH concentration at each process instant. Influence of fixed charge density ($C_{fix}$), membrane lithium concentration ($C_{Li}^{cm}$) and apparent diffusion coefficients on the membrane ($D_{Li}^{cm}\;y\;D_{OH}^{cm}$) are observed.

(9) $$J_{Li}=\frac{z_{Li}D_{Li}^{cm}C_{Li}^{cm}}{\left(D_{Li}^{cm}+D_{OH}^{cm}\right)C_{Li}^{cm}-D_{OH}^{cm}C_{fix}}\frac{i}{F}-\frac{D_{Li}^{cm}}{\Delta x}\left[\sqrt{\frac{C_{fix}^{2}}{4}+C_{LiOH}^{2}}-\sqrt{\frac{C_{fix}^{2}}{4}+{\left(C_{Li,0}^{LiCl}+\frac{V_{LiOH}}{V_{LiCl}}\left(C_{Li,0}^{LiOH}-C_{Li}^{LiOH}\right)\right)}^{2}}\right]\;\cdot \left[1+\frac{z_{Li}C_{Li}^{cm}\left(D_{OH}^{cm}-D_{Li}^{cm}\right)}{\left(D_{Li}^{cm}+D_{OH}^{cm}\right)C_{Li}^{cm}-D_{OH}^{cm}C_{fix}}\right]$$

Li^+^ concentration variation in the LiOH solution with time is calculated by Equation (10), assuming that there are both no Li^+^ losses and Li^+^ amount present in the LiCl solution volume is sufficient throughout concentration process so as not to reach depletion.

(10) $$C_{Li}^{LiOH}=C_{Li,0}^{LiOH}+\frac{N*t*A}{V_{LiOH}}J_{Li}$$

In Equation (10), N represents the number of LiOH compartments (number of basic units in the three-cell compartment), *t* is the operation time, A is the effective membrane area for each compartment and $V_{LiOH}$ is the LiOH solution volume.

Combination of Equations (9) and (10) allows for determining Li^+^ concentration variation with time, which is integrated and applied to the model.

Assumptions considered to determine Li^+^ ion transport across the cation exchange membrane are summarized below:

- Diffusion boundary layer effects are neglected.
- Linear concentration profiles in the cation exchange membrane are considered.
- Fixed charge density on membrane is determined by initial electrolyte concentrations and water content in membrane as input parameters.
- Electric current through the cation exchange membrane is carried mainly by Li^+^ ions transport as a counterion and by undesired OH^−^ transport as a co-ion.
- Solutions volume remains constant with time.

#### 2.1.2. OH^−^ Production and Cl^−^ Leakage in the Bipolar Membrane

Bipolar membrane performance within the mathematical model is developed based on work completed by Wilhelm [42], where certain assumptions are taken into account to facilitate model treatment. Among them, it is considered that H^+^ protons and OH^−^ ions generated in the bipolar membrane only transport a fraction of total current density, corresponding to the difference between applied current density and limiting current density related to salt leakage. Furthermore, diffusion boundary layer effects are neglected and it is assumed that water dissociation in the bipolar membrane has no influence on salt transport through bipolar membrane layers. According to these assumptions, H^+^ protons and OH^−^ ions fluxes generated in the bipolar membrane can be expressed mathematically by Equations (11) and (12), respectively:

(11) $$J_{H^{+}}^{prod}=\frac{i-i_{lim}}{z_{H^{+}}F}$$

(12) $$J_{OH^{-}}^{prod}=\frac{i-i_{lim}}{z_{OH^{-}}F}$$

where i is the applied current density and $i_{lim}$ is the limiting current density associated with bipolar membrane salt leakage. Thus, above limiting current density, salt leakage remains constant and the difference between applied current density value and limiting current density is proportional to H^+^ protons and OH^−^ ions production (according to Equations (11) and (12)).

Salts transport across the bipolar membrane is a function of limiting current density [13] associated with salt leakage and transport number of these salts ($t_{Li^{+}}^{lim}$,$\;t_{Cl^{-}}^{lim}$) according to Equation (13). According to the quasi-symmetric membrane concept defined by Wilhelm [42], equivalent molar fluxes of Li^+^ and Cl^−^ across the bipolar membrane are assumed; therefore, the transport number is also assumed equivalent and equal to $t_{Li^{+}}^{lim}=t_{Cl^{-}}^{lim}=0.5$.

(13) $$J_{Li^{+}}^{lim}=J_{Cl^{-}}^{lim}=t_{Li^{+}}^{lim}\frac{i_{lim}}{z_{Li^{+}}F}$$

From Equations (3) and (13), Li^+^ and Cl^−^ salt leakage through the bipolar membrane layers is related to limiting current density associated with salt leakage according to Equation (14):

(14) $$i_{lim}=2FJ_{Li^{+}}^{lim}$$

In the lithium hydroxide production process, there are concentration variations of LiOH and HCl solutions in contact with the cation exchange membrane so limiting current density changes as well. In the mathematical model, limiting current density variation associated with salt leakage according to LiOH and HCl concentration is considered according to Equation (15) based on that developed by Wilhelm [42], where solution concentration influence is observed. Thus, in the LiOH production process, bipolar membrane is influenced by both lithium hydroxide and hydrochloric acid concentration variations.

(15) $$i_{lim}=\frac{D^{bpl}F}{C_{fix}^{bpm}\Delta x_{bpl}}{\left(C_{LiOH}+C_{HCl}\right)}^{2}$$

In Equation (15), $D^{bpl}$ represents the average diffusion coefficient of salts through bipolar membrane layers, $C_{LiOH}$ is the lithium hydroxide solution concentration, $C_{HCl}$ is the hydrochloric acid solution concentration, $C_{fix}^{bpm}$ is the fixed charge density on bipolar membrane, and $\Delta x_{bpl}$ is the thickness of each bipolar membrane layer. For development of this expression, assumptions considered are summarized below:

- A quasi-symmetric membrane is assumed, where Li^+^ and Cl^−^ flux through the bipolar membrane are equivalent, but in opposite directions. Furthermore, thickness is same for both layers of the bipolar membrane $\Delta x_{a}=\Delta x_{c}=\Delta x_{bpl}$.
- Due to the continuity principle, chloride flux in the anionic layer of bipolar membrane is equal to chloride flux for the cationic layer. Same is true for lithium-ion flux.
- Linear concentration profiles in bipolar membrane layers are considered.
- Ion concentration in the intermediate catalytic region of the bipolar membrane is assumed to be zero.
- Average diffusion coefficient $D^{bpl}$, used for both transport of salts (co-ions and counterions) across anionic and cationic layers of the bipolar membrane, is defined.

Finally, LiOH production rate is determined as a function of Li^+^ flux migrating across the cation exchange membrane $\left(J_{Li}\right)$ (see Equation (9)) and OH^−^ ion concentration rate in LiOH solution $\left(J_{OH}^{LiOH}\right)$ (see Equation (16)). The latter corresponds to the difference between OH^−^ ion rate production in the bipolar membrane $\left(J_{OH}^{prod}\right)$ and OH^−^ leakage rate in the cation exchange membrane $\left(J_{OH}\right)$ and is obtained from Equations (2) and (12).

(16) $$J_{OH}^{LiOH}=J_{OH}^{prod}-J_{OH}$$

#### 2.1.3. Energy Parameters

LiOH production in a batch process is realized by aqueous concentration in parallel to HCl concentration while LiCl salt solution decreases in concentration. In an aqueous solution of a binary salt, molar conductivity ($\lambda_{m})$ depends on ionic conductivity of its components and varies with concentration according to Kohlrausch’s law (Equation (17)):

(17) $$\lambda_{m}{}^{s}=\lambda {{}^{\circ}}_{m}-K\sqrt{C^{s}}$$

where $C^{s}$ is the molar concentration of salt in aqueous state, $\lambda {{}^{\circ}}_{m}$ is the standard molar conductivity of the aqueous solution and *K* is the specific Kohlraush constant for each aqueous solution. Molar conductivity value is used to calculate specific conductivity $\left(k^{s}\right)$ and electrical resistance of solutions $\left(R^{s}\right)$ according to Equations (18) and (19), respectively:

(18) $$k^{s}=C^{s}\cdot \lambda_{m}{}^{s}$$

(19) $$R^{s}=\frac{N}{k^{s}}\cdot \frac{d}{A}$$

where *N* is the number of compartments, *d* is the distance between membranes equivalent to compartment thickness and *A* is the effective membrane area.

Additionally, electrolyte solution ($R^{elec,sol}$) and membranes’ electrical resistance ($R^{mem,a},R^{mem,c},R^{bpm}$) would depend on conductivity of solutions used and membrane specifications, respectively. Thus, cell potential difference ($\Delta E_{e}$) and overpotentials ($\eta_{a},\left|\eta_{c}\right|$) in electrolytes contribute to final cell voltage ($U_{cell}$) value in the electrodialysis stack, although to a lesser extent as the number of compartments increases. As a result, stack cell voltage is determined according to Equation (20):

(20) $$U_{cell}=\Delta E_{e}+\eta_{a}+\left|\eta_{c}\right|+i\cdot A\cdot \left(R^{HCl}+R^{LiCl}+R^{LiOH}+R^{elec\;sol}+R^{mem,a}+R^{mem,c}+R^{bpm}\right)$$

Superscripts mem,a,c y bpm refer to the membrane, anionic, cationic and bipolar, respectively.

Electrical resistance value in the bipolar membrane ($R^{bpm}$) is obtained from Equation (21), determined empirically as a function of applied current density (i) in A/m^2^ and effective membrane area (A) in m^2^. This equation has been determined from experimental data of linear sweep voltammetries (LSV) applied to a Neosepta BP membrane [37].

(21) $$R^{bpm}=\frac{2403+i}{2893\;\cdot A\;\cdot i}$$

For each simulation, specific energy consumption (SEC) is calculated according to Equation (22):

(22) $$SEC=\frac{A\;\cdot i\cdot U_{cell}\cdot t}{mp}$$

where mp is the LiOH-produced mass and t is the processing time. On the other hand, current efficiency (ϕ) is calculated according to Equation (23), based on Faraday’s law of electrolysis:

(23) $$\phi =\frac{z\cdot F\cdot V_{LiOH}\cdot \left(C_{LiOH}-C_{LiOH,0}\right)}{N\cdot A\;\cdot i\cdot t}$$

where F is the Faraday constant (96485 A·s/mol), z is the valence number, N is the number of compartments, i is the applied electric current density, A is the effective membrane area in each compartment and t is the processing time.

### 2.2. BMED Process Simulation

**Figure 2:**
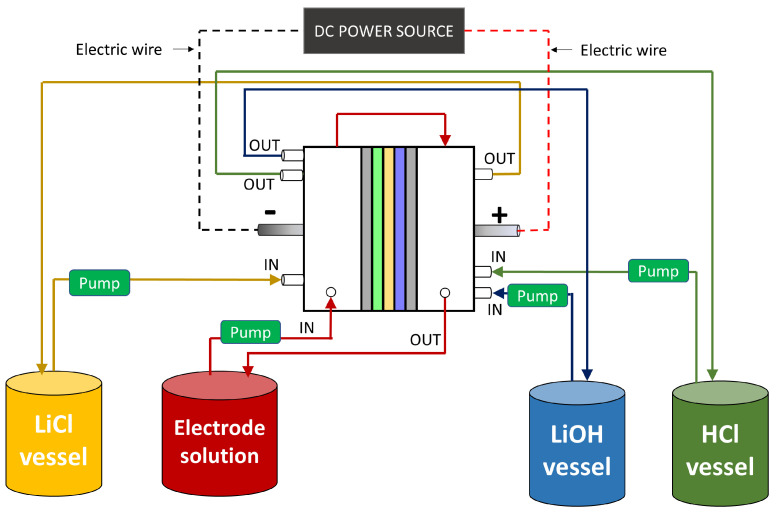
Batch process scheme for LiOH production by BMED.

For a LiOH production process by BMED parametric study, a batch configuration is used in which different solutions are recirculated in the stack. This configuration allows to analyze time-varying concentration effects of LiOH, HCl and LiCl solutions on LiOH production rate over a wide concentration range. A schematic of batch process is presented in Figure 2, consisting of three main streams with their respective vessels. Additionally, an electrode solution with a constant conductivity of 100 mS/cm is used. Its objective is to provide conductivity in the electrode compartment and at the same time avoid chlorine gas (Cl_2_) formation, so that its concentration throughout the process is considered constant.

Membrane characteristics such as ion exchange capacity, fixed charge density, water content, thickness and diffusion coefficients in conjunction with electrode compartment characteristics used in the model are presented in Table 1. Ion exchange membrane charge density is determined according to Equation (24) [50], assuming an ion exchange capacity value of 1.6 meq/gr for all cases in the parametric study.

(24) $$C_{fix}=\frac{IEC\cdot \rho_{w}}{w_{u}}$$

where IEC is the ion exchange capacity, $\rho_{w}$ is the density of water at 25 °C and $w_{u}$ is the membrane water percentage content.

Regarding membrane water content, values are assumed according to initial solutions concentration used, obeying the tendency to decrease with increasing concentration [37,51]. Thus, from previous studies [37], for initial LiCl concentrations of 14, 25 and 34% by mass, water content in the membrane of 33.5%, 31.5% and 29.7%, respectively, is established.

On the other hand, in order to evaluate different degrees of OH^−^ leakage in cationic membranes, the effect of different apparent diffusion coefficients [52] for OH^−^ ion ($D_{OH}^{cm}$) with values between 3∙10^−12^ m^2^/s and 27∙10^−12^ m^2^/s is analyzed, assuming different resistance to OH^−^ ion transport in cation exchange membranes. For this purpose, an apparent diffusion constant Li^+^ ion coefficient with a value of 9.0∙10^−12^ m^2^/s is established. In addition, for the bipolar membrane in order to determine its influence on the process, different average salt diffusion coefficients ($D^{bpl}$) between 60∙10^−12^ and 140∙10^−12^∙m^2^/s and fixed charge densities ($C_{fix}^{bpm}$) between 3500 and 6500 mol/m^3^, corresponding to ion exchange capacities of approximately 0.90 and 1.65 meq/gr, respectively, are considered.

Finally, electrode compartment voltage drop is considered constant, having less influence on electrodialysis stacks as the number of compartments increases [53]. Thus, in electrode compartments, electrode potential corresponding to O_2_ and H_2_ formation at anode and cathode, respectively, is considered constant. On the other hand, overpotentials are assumed constant during the whole process with a value of 0.1 V.

#### 2.2.1. Validation

To validate the model, simulated results were compared with experimental results of LiOH production by BMED at a laboratory scale [37]. Data were obtained from experimental tests using a bipolar membrane electrodialysis stack with an effective membrane area of 27.5 cm^2^ (55 mm × 50 mm) and a membrane spacing of 1.0 mm. CMX and CMB membranes (Tokuyama Corp, Tokyo, Japan) were used as cation exchange membranes, AMX (Tokuyama Corp, Japan) as anionic membranes, and Neosepta BP-1 and Fumasep FBM as bipolar membranes. For the preparation of aqueous solutions, lithium chloride (LiCl) was purchased from Winkler (Winkler ltda, Santiago, Chile), while lithium hydroxide (LiOH) and hydrochloride acid (HCl) were purchased from Merck (Merck, Darmstadt, Germany). The purity of all reagents was ≥99%.

Validation was performed considering four configurations presented in Table 2, using initial concentrations of 210 mol/m^3^ LiOH (approx 0.5% by mass) and 0.137 mol/m^3^ HCl (approx 0.5% by mass) with volumes of 450 mL for LiOH and HCl solutions.

#### 2.2.2. Parametric Study

For development of the parametric study, a hypothetical stack design with specific characteristics and operating conditions presented in Table 3 is considered. Subsequently, process sensitivity to variation of certain parameters such as applied current density, concentration, solution volume and number of compartments is evaluated. All simulations are performed at room temperature (25 °C) in order to avoid adding more energy consumption to the process.

##### OH^−^ Leakage in Cationic Membrane Evaluation

In the present study, in order to quantify process efficiency sensitivity to OH^−^ ion leakage, the process is simulated using data from Table 1 and Table 3. Three different apparent diffusion coefficients for OH^−^ ion with values of 3∙10^−12^, 9∙10^−12^ and 27∙10^−12^ m^2^/s are considered. Thus, three different degrees of OH^−^ leakage are simulated, with an apparent diffusion coefficient of OH^−^ greater than, equal to and less than the apparent diffusion coefficient of Li^+^ ion (9∙10^−12^ m^2^/s). In practice, this is defined by cationic membranes' specific characteristics. For this comparison, an initial 6800 mol/m^3^ LiCl concentration and a 1000 A/m^2^ current density are used. On the other hand, for the bipolar membrane, a fixed charge density of 6500 mol/m^3^ and a diffusion coefficient of 1.4∙10^−10^ m^2^/s are configured.

##### Bipolar Membrane Performance Evaluation

Regarding bipolar membrane performance, process sensitivity to variations of fixed charge density of its layers $\left(C_{fix}^{bpm}\right)$ and of average diffusion coefficients related to salt leakage $\left(D^{bpl}\right)$ is analyzed. From bipolar membrane characteristics presented in Table 1, process sensitivity to fixed charge density $\left(C_{fix}^{bpm}\right)$ is tested over a range of 3500 to 6500 mol/m^3^ for a constant average membrane diffusion coefficient of 1.4 × 10^−10^ m^2^/s. Subsequently, sensitivity to average diffusion coefficient $\left(D^{bpl}\right)$ is tested over a range of 0.6 × 10^−10^ to 1.4 × 10^−10^ m^2^/s for a constant fixed charge density of 6500 mol/m^3^. These conditions are simulated at an initial LiCl concentration of 6800 mol/m^3^ and a current density of 1000 A/m^2^, while an apparent OH^−^ diffusion coefficient of 27 × 10^−12^ m^2^/s is considered in the cation exchange membrane.

##### Sensitivity to Operating and Design Conditions

For parametric study development, we studied the effect of current density $\left(i\right)$ in the range of 300 to 2000 A/m^2^ on LiOH production. For this purpose, LiOH production tests were simulated at an initial LiCl concentration of 6800 mol/m^3^ considering as reference design and operating conditions in Table 3.

Regarding electrolyte concentration, three different initial concentrations of LiCl (3600, 6800 and 9600 mol/m^3^) were simulated at a current density of 1000 A/m^2^, evaluating their effect on LiOH production rate, Cl^−^ ion leakage in the bipolar membrane, current efficiency and specific electrical electricity consumption (SEC).

Regarding stack design, effect of the number of LiOH compartments in a range of 10 to 100 basic units of three-cell compartments was evaluated. These simulations provided information regarding influence of total membrane area for the same volume of LiOH that is recirculated. Thus, the ratio between total membrane area and volume of LiOH solution is in a range between 12 to 120 m^2^ of membrane per m^3^ of LiOH solution. These conditions were simulated at an initial LiCl concentration of 6800 mol/m^3^ and a constant current density of 1000 A/m^2^.On the other hand, effect of the ratio between treated volumes of HCl and LiOH that were processed ($V_{LiOH}/V_{HCl}$) was analyzed. This ratio variation allowed for analysis of different LiOH and HCl concentration ratios that could coexist in a specific process time and their effect on performance. For the study, $V_{LiOH}/V_{HCl}$ ratios between 1.0 and 0.1 were simulated, i.e., according to Table 3 for a LiOH solution volume of 0.025 m^3^, the hydrochloric acid solution volume was varied between 0.025 and 0.25 m^3^, respectively. These conditions were simulated at an initial LiCl concentration of 6800 mol/m^3^ and a constant current density of 1000 A/m^2^.

## 3. Results

### 3.1. OH^−^ Leakage

**Figure 3:**
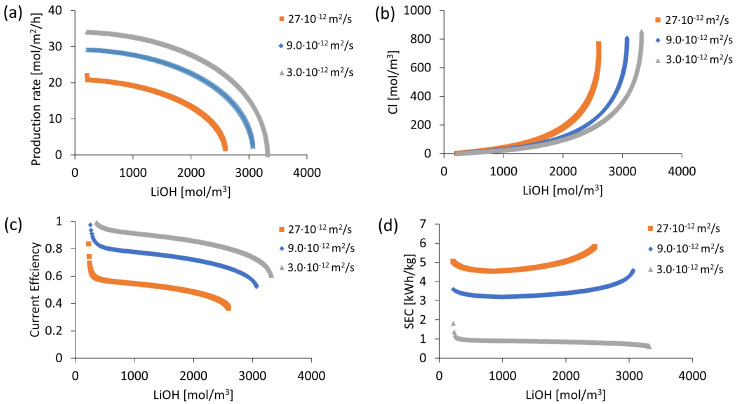
Simulation results according to different degrees of OH^−^ leakage: (**a**) LiOH production rate; (**b**) Cl^−^ concentration; (**c**) Current efficiency; (**d**) Specific electricity consumption (SEC).

In the cation exchange membrane, OH^−^ transport is different from other co-ions due to its high ionic mobility. Therefore, OH^−^ ion leakage in the cation exchange membrane was simulated by considering different apparent OH^−^ ion diffusion coefficients. Results in Figure 3a showed the influence of this parameter on LiOH production capacity, reaching LiOH production process beginning rates of 34, 29 and 22.0 mol/m^2^/h when using OH^−^ diffusion coefficients of 3 × 10^−12^, 9 × 10^−12^ and 27 × 10^−12^ m^2^/s, respectively. General process behavior corresponded to gradual LiOH solution concentration at a specific production rate for each diffusion coefficient with decreasing concentration until reaching zero, stopping production. It was also observed in Figure 3b that when reaching high concentrations of LiOH, there was higher contamination with Cl^−^ ion due to its leakage in the bipolar membrane. However, a lower OH^−^ diffusion coefficient allowed the LiOH production rate to be higher than Cl^−^ leakage rate, reducing Cl^−^ contamination. That was the case when a concentration of 2500 mol/m^3^ of LiOH was reached, where Cl^−^ concentration for the apparent OH^−^ diffusion coefficient value of 3 × 10^−12^ m^2^/s was 58.6% lower than that obtained with a value of 27 × 10^−12^ m^2^/s, with values of Cl^−^ concentrations of 183.5 mol/m^3^ and 442.2 mol/m^3^, respectively.

On the other hand, current efficiency variation and specific electricity consumption were presented in Figure 3c,d, respectively. It was observed that both parameters highly benefited from lower OH^−^ leakage. In the best case, with an apparent OH^−^ diffusion coefficient of 3 × 10^−12^ m^2^/s, a current efficiency higher than 0.8 and an approximate SEC of 1.0 kWh/kg was achieved up to a 2622 mol/m^3^ LiOH concentration without increasing the SEC value. The latter corresponded to an optimistic case in which the use of a hypothetical high-efficiency cation exchange membrane resistant to OH^−^ ion leakage was assumed. However, when comparing simulation results with experimental data, it was found that actual behavior is best fit by using an apparent diffusion coefficient between 27 × 10^−12^ m^2^/s and 35 × 10^−12^ m^2^/s. This value of the apparent diffusion coefficient of OH^−^, which was even higher than that of Li^+^ ion, better represented actual OH^−^ ion leakage in the cation exchange membrane. This could be explained by high OH^−^ ion ionic mobility compared to other anions. Therefore, the process parametric study was performed using a value of 27 × 10^−12^ m^2^/s for OH^−^ ion as the apparent diffusion coefficient.

### 3.2. Process Sensitivity to Bipolar Membrane Performance

**Figure 4:**
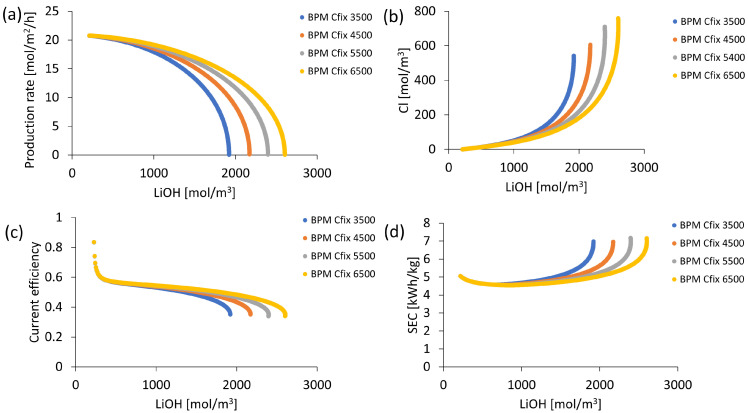
Process performance according to fixed charge density in the bipolar membrane: (**a**) LiOH production rate; (**b**) Cl^−^ concentration; (**c**) Current efficiency; (**d**) Specific electricity consumption (SEC).

**Figure 5:**
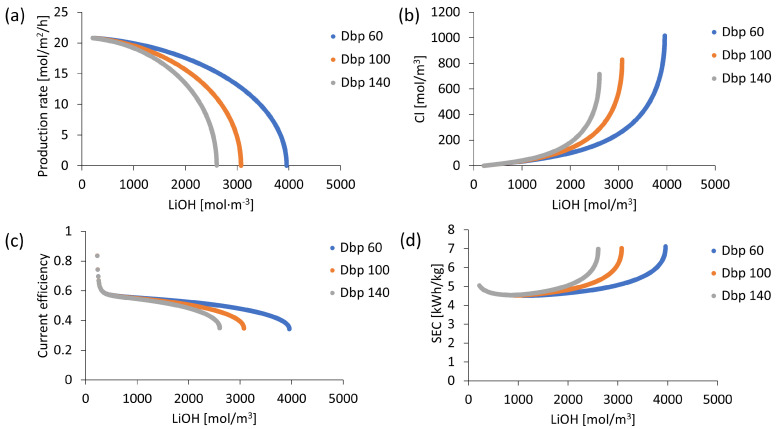
Process performance according to the average diffusion coefficient in the bipolar membrane ranges from 60 × 10^−12^ to 140 × 10^−12^ m^2^/s (Dbp 60 to Dbp 140 in the graph, respectively): (**a**) LiOH production rate; (**b**) Cl^−^ concentration; (**c**) Current efficiency; (**d**) Specific electricity consumption (SEC).

Process sensitivity to bipolar membrane performance was studied by varying its fixed charge density and average diffusion coefficients. Results were presented in Figure 4 and Figure 5, respectively. Bipolar membrane performance was mainly determined by limiting current density associated with salt leakage. It could be inferred from Equation (15) that the limiting current density was lower at high values of fixed charge density ($C_{\mathrm{fix}}^{\mathrm{bpm}}$) and low values of the average diffusion coefficient ($D^{\mathrm{bpl}}$). In Figure 4a, when comparing results for a fixed charge density of 3500 and 6500 mol/m^3^, when a LiOH solution concentration of 1800 mol/m^3^ (approximately 4.14% by mass) was reached, LiOH production rates of 7.65 and 15.04 mol/m^2^/h, respectively, were obtained. For such a LiOH concentration, chloride contamination was 53% lower when a fixed loading density of 6500 mol/m^3^ was used (see Figure 4b). On the other hand, for fixed charge densities between 3500 and 6500 mol/m^3^ on the bipolar membrane, maximum LiOH concentrations of 1920 and 2600 mol/m^3^ were achieved, equivalent to approximately 4.33% and 5.74% by mass, respectively. Regarding current efficiency and specific electrical energy consumption (SEC) in Figure 4c,d, respectively, it was observed that below a 1000 mol/m^3^ LiOH concentration a similar behavior was observed with an approximate current efficiency of 0.6 and an SEC close to 5 kWh/kg. Above this concentration, process energy efficiency showed differences in performing better for high values of fixed charge density ($C_{\mathrm{fix}}^{\mathrm{bpm}}$). This was related to LiOH production rate variation.

Regarding the average diffusion coefficient of salts in the bipolar membrane ($D^{\mathrm{bpl}}$), a high influence on the maximum LiOH concentration that could be reached was observed in Figure 5a. By using values of 60 × 10^−12^, 100 × 10^−12^ and 140 × 10^−12^ m^2^/s, maximum LiOH concentrations of 3955, 3073 and 2603 mol/m^3^, respectively, were achieved. This was equivalent to an approximate mass concentration range between 8.44% and 5.74%. Average diffusion coefficients lower than 140 × 10^−12^ m^2^/s allowed for reaching LiOH concentrations higher than 2600 mol/m^3^ with low Cl^−^ content (see Figure 5b), reducing the increase in specific electrical energy consumption at such concentrations. It was observed in Figure 5c,d, that upon reaching a 1000 mol/m^3^ LiOH concentration, in all cases a current efficiency and SEC close to 0.55 (50%) and 4.5 kWh/kg were obtained, respectively.

Both fixed charge density and average diffusion coefficient in the bipolar membrane influence the LiOH production rate. However, their different magnitudes' effects increase at high concentrations. It is then determined that fixed charge density and average diffusion coefficients define bipolar membrane performance at high LiOH concentrations and depend on specific membrane characteristics determined by its functional groups and the polymeric matrix.

### 3.3. Model Validation

Given process sensitivity results to OH^−^ leakage and bipolar membrane performance, values of diffusion coefficients and fixed charge density to be used in the validation of the model were defined. Thus, for the cation exchange membrane, an apparent diffusion coefficient of Li^+^ and OH^−^ ion of 9 × 10^−12^ and 35 × 10^−12^ m^2^/s, respectively, was used. An ion exchange capacity of 1.6 and 2.4 meq/gr [37], respectively, was used for CMX and CMB membranes. While for the bipolar membrane, the best match with experimental data was obtained with an average diffusion coefficient of 140 × 10^−12^ m^2^/s. According to bipolar membrane water content data [37], for the Neosepta BP membrane, an average fixed charge density of 6004 mol/m^3^ was estimated by fitting. Similarly, for the Fumasep FBM membrane, a value of 5260 mol/m^3^ was obtained by fitting.

Mathematical model validation considered four configurations which differ mainly in applied current density, initial lithium chloride concentration and the number of cells. Configuration 1 was subdivided into Configuration 1F and Configuration 1N, referring to the use of bipolar membranes Fumasep FBM and Neosepta BP, respectively. Similarly, configuration 3 was subdivided into Configuration 3X and 3B, referring to the use of cationic membranes CMX and CMB, respectively. Configurations 2 and 4 used cationic CMX membranes and bipolar membrane Fumasep FBM.

**Figure 6:**
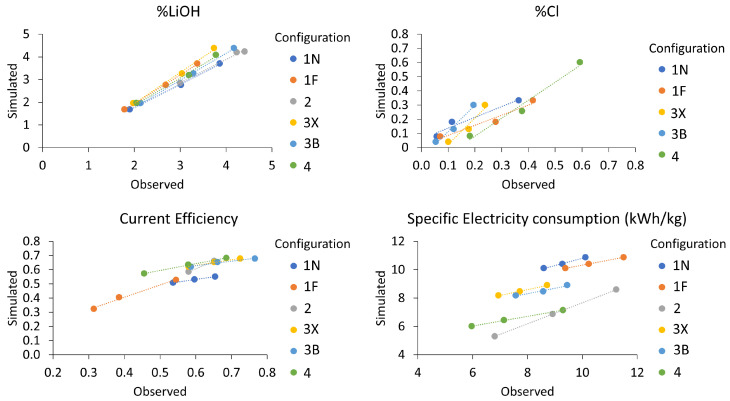
Comparison of observed and predicted values of LiOH concentration, Cl^−^ concentration, current efficiency and SEC.

Figure 6 compares observed and predicted values of LiOH concentration, Cl^−^ concentration, current efficiency and specific power consumption. Graphs indicate a good qualitative prediction between different configurations. For example, the model correctly predicts that configuration 4 would present the highest Cl^−^ ion contamination, that configuration 2 would present the lowest current efficiency (CE) and that the highest SEC is presented by configuration 1F, among other similarities. However, it can be seen that when comparing observed and predicted values, not all lines intercept the origin, implying the existence of certain deviations.

Determination coefficients R^2^ and associated root-mean-square error (RMSE) values are presented in Table 4, comparing the correlation between observed and predicted values of different output parameters such as LiOH concentration, Cl^−^ concentration, current efficiency and purity, among others. Most R^2^ coefficients are in the range of 93.116% to 99.9999%. The largest variation in root-mean-square error (RMSE) is presented by SEC and voltage, which can be attributed to changes in membrane electrical resistance not considered in the model (fouling or sudden variations in water content), and deviations associated with solutions electrolytic conductivity calculation in a wide range of concentration or other considered assumptions.

Based on the above, it is determined that the model satisfactorily predicts LiOH production process behavior for purpose of a parametric study.

### 3.4. LiOH Production According to Electric Current Density

**Figure 7:**
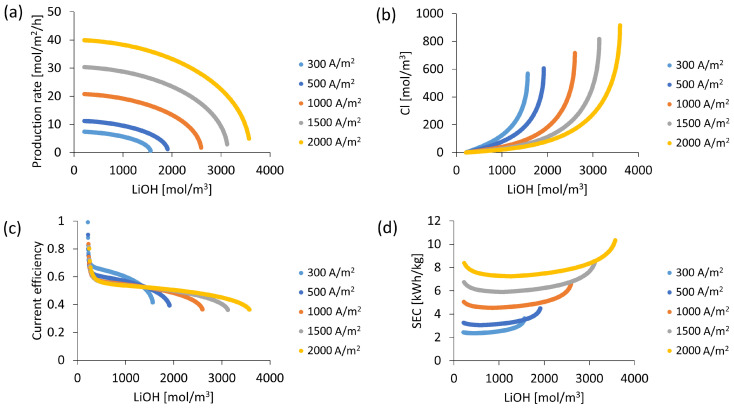
Process performance according to electric current density applied at an initial concentration of LiCl 6800 mol/m^3^: (**a**) LiOH production rate; (**b**) Cl^−^ concentration; (**c**) Current efficiency; (**d**) Specific electricity consumption (SEC).

From the stack design and operating conditions defined in Table 3, the effect of electric current density on the LiOH production process is analyzed. Figure 7 presents LiOH production rate variation, Cl^−^ ion contamination, current efficiency and specific electrical power consumption as the LiOH solution is concentrated.

Applied electric current density has a direct effect on the maximum LiOH concentration that can be achieved. It is observed in Figure 7a that when using current densities between 300 and 2000 A/m^2^ the maximum LiOH concentrations achieved vary between 1566 mol/m^3^, and 3594 mol/m^3^, respectively (approximately 3.6% and 7.7% by LiOH mass).

Regarding Cl^−^ ion leakage in the bipolar membrane in Figure 7b, it decreases with increasing applied electric current density as related to a greater difference with limiting current density in the bipolar membrane associated with salt leakage. That is, more electric current is used in the generation of OH^−^ ions over undesired Cl^−^ migration. Comparing results at 2000 A/m^2^ and 300 A/m^2^, a higher current density value allows us to reach approximately twice the LiOH concentration with a similar Cl^−^ content as the contaminant. Therefore, for the same LiOH concentration, a high current density reduces Cl^−^ concentration in the final solution.

On the other hand, in relation to energetic parameters, an electrical current density lower than 300 A/m^2^ allows us to achieve current efficiencies between 0.6–0.7(60–70%) up to an approximate LiOH concentration of 992 mol/m^3^ (approx 2.3% by mass). From this concentration, current efficiency decreases in an accelerated manner to 0.4 (40%) (see Figure 7c). However, current densities greater than 1000 A/m^22^ allow higher LiOH concentrations slowing down current efficiency reduction, for these cases, with current efficiencies in the 0.5–0.6 (50–60%) range.

Specific electricity consumption (SEC) increases with current density (see Figure 7d). When using values between 300 and 500 A/m^2^, SEC remains most of the process between 2 and 4 kWh/kg. Furthermore, for current densities of 1000, 1500 and 2000 A/m^2^ the average SEC obtained increases to 5.6, 7.4 and 9.1 kWh/kg, respectively.

In all cases, during LiOH solution concentration the minimum SEC is observed. Then, when approaching maximum concentration, SEC increases rapidly while current efficiency decreases. The minimum SEC value would also depend on other factors such as solution concentration and stack design. Higher current densities would contribute to higher final concentration driving a higher LiOH production rate.

### 3.5. Initial Concentration Influence

**Figure 8:**
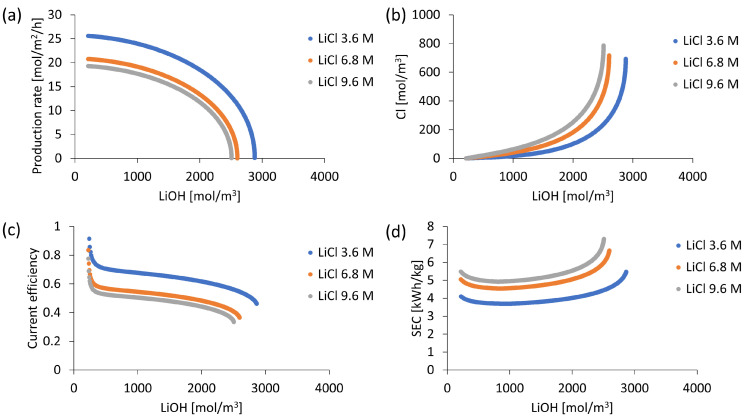
Simulation results for three different LiCl concentrations at a current density of 1000 A/m^2^: (**a**) LiOH production rate; (**b**) Cl^−^ concentration; (**c**) Current efficiency; (**d**) Specific electricity consumption (SEC).

LiCl and LiOH solutions establish a concentration gradient that serves as a driving force in Li^+^ transport across the cation exchange membrane. In addition, aqueous solution concentration affects membrane stack electrical conductivity. The effect of such characteristics on the production and energy efficiency of the BMED process is quantified by considering three different initial concentrations of LiCl with corresponding results presented in Figure 8. While high LiCl concentration causes a higher concentration gradient driving lithium transport, it is observed that the LiOH production rate decreases at higher initial LiCl concentration (see Figure 8a), which can be explained due to the increase of Li^+^ counterions concentration in the cationic membrane. According to the electroneutrality principle, a large difference of Li^+^ concentration in the cation exchange membrane with fixed charge density (C_fix_) results in a high concentration of co-ions, promoting OH^−^ leakage according to the Nernst–Planck equation (Equation (2)). Compared to a 3600 mol/m^3^ LiCl solution, when using a 6800 mol/m^3^ solution, the counterions concentration in the membrane is 16% higher while co-ions concentration is 181% higher. Furthermore, when using a 9600 mol/m^3^ solution, counterions and co-ions concentration in the membrane is 33% and 384% higher, respectively. This causes a molar Li^+^ flux in the cation exchange membrane to decrease for high LiCl concentrations. The simulation indicates that throughout the LiOH concentration process, for LiCl concentrations 3600, 6800 and 9600 mol/m^3^, the average Li^+^ transport number in the cation exchange membrane corresponds to 0.66, 0.53 and 0.50, respectively. Thus, during the LiOH concentration process by using an initial concentration of 3600 mol/m^3^ LiCl, the LiOH production rate is 23% higher compared to 6800 mol/m^3^ and 33% higher compared to 9600 mol/m^3^. On the other hand, higher LiCl concentration favors undesired Cl^−^ ion transport into the LiOH compartment due to its diffusion across the cation exchange membrane in order to reach electrical neutrality in solution (see Figure 8b). That is, for high LiOH concentrations, the lithium migration rate into the LiOH compartment is higher than OH^−^ concentration rate (Equation (16)) increasing undesired Cl^−^ transport from the LiCl compartment.

Regarding current efficiency presented in Figure 8c, the best result is obtained when working with a LiCl concentration of 3600 mol/m^3^, which allowed a current efficiency higher than 0.7 (70%) upon reaching a concentration of 556 mol/m^3^ (approximately 1.3% by mass) and higher than 0.6 (60%) up to a LiOH concentration of 2181 mol/m^3^ (approximately 5.0% by mass). These current efficiency values relate to a high production rate achieved at 3600 mol/m^3^ LiCl concentration.

**Figure 9:**
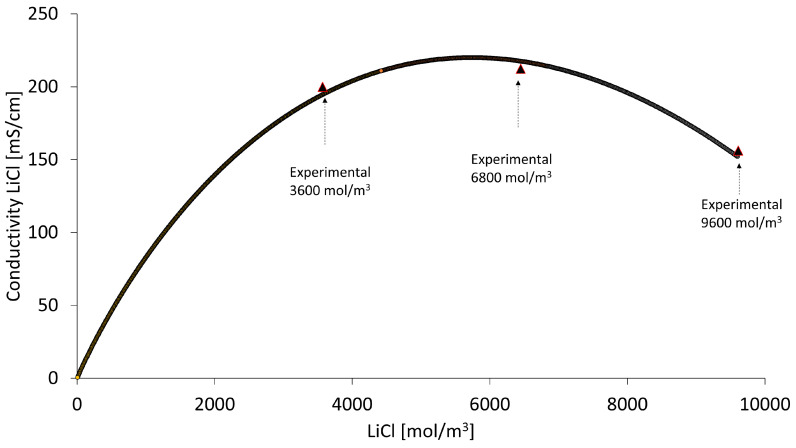
Electrolytic conductivity variation of LiCl with concentration.

The mathematical model delivers simulated electrolytic conductivity data for solutions used with LiCl solution being particularly influential on total stack voltage. Figure 9 shows that the highest electrical conductivity of LiCl is achieved when working at a LiCl concentration of 6800 mol/m^3^ (approximately 25 % by mass) and is lower as the solution is diluted. Despite this, the resulting high production rate at a concentration of 3600 mol/m^3^ allows the lowest specific electrical energy consumption (SEC) to be obtained compared to higher LiCl concentrations, averaging 4.3 kWh/kg over a range of 3.7 to 5.8 kWh/kg (see Figure 8d).

### 3.6. Influence of Number of Compartments

Increasing the number of compartments in an electrodialysis stack has direct effects on electrical resistance and total voltage drop.

**Figure 10:**
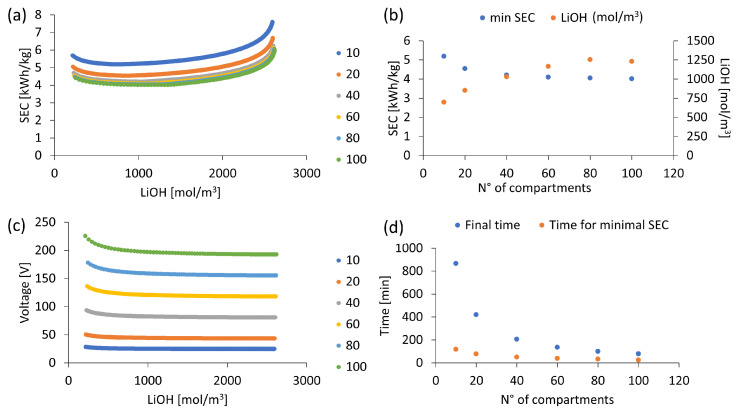
Number of compartments effect. Current density of 1000 A/m^2^, initial concentration 210 mol/m^3^ of LiOH: (**a**) Specific electricity consumption; (**b**) Specific minimum SEC for a corresponding LiOH concentration; (**c**) Voltage variation with concentration; (**d**) Final operating time vs time for minimum SEC.

Figure 10a,c present specific electrical energy consumption (SEC) variation and total voltage drop in the stack according to the number of compartments in a range from 10 to 100 basic units three-cell compartments, respectively. It is observed that voltage increases and SEC decreases with the number of compartments. More compartments imply a larger total effective membrane area available for Li^+^ transport and LiOH formation. This higher production rate causes a tendency to reach a minimum SEC close to 4 kWh/kg when using 100 compartments. Increasing from 20 to 40 compartments reduces SEC by 7.3% while increasing from 80 to 100 compartments implies a reduction of SEC by 0.8%. Therefore, the average specific electrical energy consumption (SEC) decreases with the number of compartments. However, beyond 80 compartments, the decrease in SEC is less than 1%.

Figure 10b shows that for each compartment number configuration, a specific minimum SEC is obtained for a corresponding LiOH concentration. As the compartment number increases, the minimum SEC decreases, and its corresponding LiOH concentration increases. Likewise, as the effective membrane area is increased, it is possible to achieve the desired LiOH concentration levels in less time.

Figure 10d shows that for a target LiOH concentration close to 2570 mol/m^3^ (approx. 5.8% by mass), increasing from 40 to 60 compartments allows a reduction in process time by 34.5% (from 206 to 135 min). Then, increasing from 60 to 80 and from 80 to 100 compartments reduces time by 25.9% and 21%, respectively.

In conclusion, increasing the number of compartments reduces process time and specific electrical energy consumption (SEC) and increases the LiOH concentration at which the minimum SEC is obtained. However, these advantages are reduced beyond 80 compartments. Regarding LiOH production rate and Cl^−^ leakage in the bipolar membrane, results show no significant differences with the number of compartments, these parameters being mainly dependent on concentration, current density and membrane characteristics.

### 3.7. Initial Solution Volume Variation

**Figure 11:**
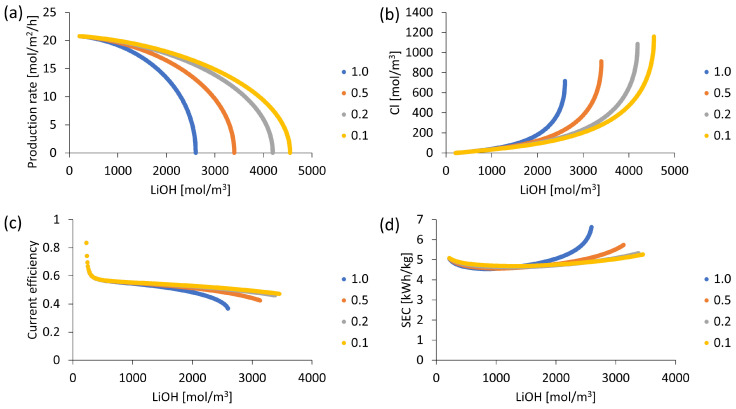
Treated volume ratio effect of HCl and LiOH. Used 6800 mol/m^3^ LiCl and 210 mol/m^3^ LiOH, current density 1000 A/m^2^: (**a**) LiOH production rate; (**b**) Cl^−^ concentration; (**c**) Current efficiency; (**d**) Specific electricity consumption (SEC).

It has been observed that LiCl concentration affects cation exchange membrane performance and HCl concentration affects limiting current density associated with salt leakage in the bipolar membrane (according to Equation (15)). Therefore, it follows that keeping LiCl and HCl concentrations within certain ranges can contribute to controlling unwanted phenomena such as Cl^−^ ion leakage and improve process performance, which could be conducted by controlling volumes used in each batch. Figure 11 shows the performance results of BMED process for different volume ratios $V_{LiOH}/V_{HCl}$ in a range from 0.1 to 1.0. It is observed from Figure 11b that a lower value of $V_{LiOH}/V_{HCl}$ ratio helps to reduce Cl^−^ ion leakage in the bipolar membrane, and, as a consequence, a higher LiOH production rate is obtained. This is attributable to the dependence of salt leakage limiting current density on solution concentration in contact with bipolar membrane (Equation (15)). Figure 11a,b show that by using a $V_{\mathrm{LiOH}}/V_{\mathrm{HCl}}$ the ratio of 0.1, it would be possible to obtain a LiOH concentration between 29–30% higher compared to a 1.0 ratio, with a Cl^−^ concentration less than 100 mol/m^3^ (0.33% by mass) in both cases.

Unwanted phenomena such as OH^−^ leakage into the LiCl compartment and Cl^−^ leakage from the HCl solution to the LiOH compartment limit LiOH production and final concentration in the solution. Cl^−^ ion leakage consumes electrical energy by reducing OH^−^ formation in the catalytic region of the bipolar membrane. Maintaining a low HCl concentration helps to reduce Cl^−^ ion leakage into the bipolar membrane and consequently increases process efficiency. Thus, it is observed in Figure 11d that when working with a $V_{\mathrm{LiOH}}/V_{\mathrm{HCl}}$ the ratio of 0.1, specific electrical energy consumption (SEC) is maintained between 4.68 and 5.04 kWh/kg up to LiOH concentrations close to 3000 mol/m^3^ (approximately 6.7% by mass). In addition, electric current efficiency presents higher stability (always higher than 0.5) (see Figure 11c).

Regarding the most optimal case, when comparing a $V_{\mathrm{LiOH}}/V_{\mathrm{HCl}}$ the ratio of 0.1 and 0.2 for a target concentration of 2000 mol/m^3^ LiOH, energy efficiency curves (SEC and CE) almost overlap presenting very close values. The use of a $V_{\mathrm{LiOH}}/V_{\mathrm{HCl}}$ ratio of 0.2 allows a 1.9% lower LiOH production rate compared to a 0.1${\;V}_{\mathrm{LiOH}}/V_{\mathrm{HCl}}$ ratio.

The use of a larger volume of HCl solution allows for reducing Cl^−^ leakage in the LiOH concentration process. However, its impacts on other stages of the overall process on a larger scale, related to its water consumption and costs, should be investigated.

## 4. Discussion and Application Potential

The mathematical model developed allows for estimating LiOH production and its performance parameters for different operating conditions, stack design and process design. The influence of phenomena such as OH^−^ leakage in the cation exchange membrane and Cl^−^ leakage in the bipolar membrane at high concentrations has been quantified for different operating conditions. The presented model provides important information through process performance estimates, validated over a wide range of concentration and operating conditions.

Efficient production requires low salt leakage in the bipolar membrane and low OH^−^ leakage in the cation exchange membrane. For the application of this technology in LiOH production, this efficiency would depend on the characteristics of the ion exchange membranes used. It is determined that the use of improved hypothetical ion exchange membranes with a low OH^−^ ion leakage increases production efficiency by approximately 20%, improving product solution purity. Given the scope of current technology, both the experimental results presented in Table 5 and the results obtained in this mathematical model show that it is possible to obtain a purity of LiOH in a solution of 95% by mass. This solution purity allows for obtaining high-purity LiOH·H_2_O crystals by evaporative crystallization processes.

A comparison of LiOH production yield results using Chlor-Alkali/Membrane electrolysis and BMED technologies is presented in Table 5. Experimental results found in the literature show that depending on initial feed concentration and final LiOH solution concentration the specific electricity consumption varies between 3.4 and 14.6 kWh/kg of LiOH, while the current efficiency varies between 14% and 77%. These values are within the ranges obtained in the different simulations performed in the present study.

In this work, the mathematical model shows that it is possible to reduce the specific electricity consumption by developing better membranes, resistant to salt leakage. Simulations performed suggest that LiOH production by BMED should focus on obtaining LiOH concentrations between 1700 and 2000 mol/m^3^ (approx 4.0–4.5% by mass) of LiOH, which implies a specific electricity consumption between 3.9 and 4.0 kWh/kg. This value is lower compared to what has been reported experimentally [37,38,39]. This occurs when using an initial LiCl concentration of 3600 mol/m^3^ and an electric current density no greater than 1000 A/m^2^ (see Figure 8). Above these LiOH concentrations, process efficiency and solution purity decrease. On the other hand, specific electricity consumption decreases with decreasing LiCl feed concentration. It has been shown that for a LiCl concentration range of 70–130 g/L (approximately 7–12% by mass), specific electricity consumption is less at 130 g/L [38]. The latter, in conjunction with the results of this work, suggests an optimal initial LiCl concentration between 12 and 14%, which can be explained by electrolytic conductivity variation (see Figure 9).

In practice, limiting current densities that promote concentration polarization in monopolar membranes and damage to the bipolar membrane associated with low water diffusion into the catalytic intermediate zone should be avoided [54]. The bipolar membrane must be stable during the operating conditions used. Damage to the catalytic intermediate zone at high current densities must be avoided by ensuring diffusive water transport in membrane layers [43]. Regarding the cation exchange membrane, depending on its structure and composition, these can be affected by contact with OH^−^ ions. It has been reported that they may suffer a loss of functional groups and damage to the polymeric matrix or their backing textile structure [42,55,56]. For the concentration of bases, the use of membranes manufactured on the basis of perfluorinated base polymers is suggested, which carry sulfonic acid groups and a layer with carboxylic acid groups in contact with the alkaline solution, reducing water transport by limiting OH^−^ leakage [42,57].

Regarding the effect of impurities present in brines on process performance, prior purification of brines is always necessary in order to reduce cations concentration of other than lithium to a minimum. Specifically, multivalent cations such as calcium and magnesium must be removed, as they can cause complex formation in the fixed charge resulting in membrane scaling and poisoning [58,59]. The mathematical model presented assumes an initial lithium chloride brine free of impurities. Calcium and magnesium removal can be achieved by combining chemical precipitation and ion exchange technologies, reducing these cations to trace levels in concentrated LiCl solutions greater than 29% by mass [60]. Other direct lithium extraction technologies would allow obtaining LiCl solutions at low concentrations free of impurities that would be suitable for application to a BMED process [61,62]. Other monovalent cations such as Na^+^ and K^+^ should be reduced to the minimum possible because they compete with Li^+^ in migration across the cation exchange membrane, reducing their transport number, process efficiency and product solution purity. These cations exhibit higher diffusion in cation exchange membranes compared to lithium due to their lower hydration energy [63]. However, their transport across membranes can be reduced if they are at low concentrations compared to other cations [64]. For high process performance, it is necessary to ensure an initial feed brine in a suitable concentration range (12–14% by mass) low in impurities, which in addition to affecting membranes could require modifications to overall process design demanding post-removal, or increased purge or water consumption.

**Table 5:** Comparison of LiOH production yield results using Chlor-Alkali/Membrane electrolysis and BMED technologies.

Technology	Initial Concentration	Final LiOH Concentration	Final LiOH Solution Purity	SEC (kWh/kg of LiOH)	Current Efficiency	Ref.
Chlor-Alcali/Membrane electrolysis	13.4% by mass LiCl	-	-	7.25	70%	[11]
	19.1% by mass Li_2_SO_4_	3% by mass	-	6.1–14.6	45–70%	[65]
BMED	14% by mass LiCl	4.05–4.35%	95.4%	8.71–9.45	77–59%	[37]
	70–130 g/L LiCl (approx 6.7–12.0% by mass)	1.50–1.75 M (approx 3.5–4.0% by mass)	-	Approx 3.4–4.0	Approx 50–60%	[38]
	1.5 M Li_2_SO_4_	2.2 M (approx 5.0% by mass)	-	10	55%	[39]
	0.05 M Li_2_SO_4_	0.9 M (approx 2.1% by mass)	99.75%	7	74%	
	60–120 g/L LiCl (approx 5.8–11.3% by mass)	1.73–2.69 M (approx 3.99–6.07% by mass)	-	5.51–8.96	24.01–14.07%	[66]
Simulated BMED	14% by mass	Approx 4.0–4.5% by mass	>95%	3.9–4.0	60–80%	This work

## 5. Conclusions

Through the mathematical model, LiOH production by BMED was simulated at high concentrations of LiCl by testing the effect of various parameters on the LiOH concentration process. Given the current characteristics of ion exchange membranes, working with high electrolyte concentrations favors the undesired transport of salts. The current scope of technology allows the best result to obtain LiOH solutions greater than 4% by mass with a purity of 95% without compromising a high purity of the LiOH·H_2_O obtained after a crystallization step.

According to the simulations performed, a high current density allows a higher LiOH production rate and lower Cl^−^ ion leakage in the bipolar membrane. The best energy efficiency is obtained with an electric current density of less than 1000 A/m^2^ and when working with a LiCl concentration of 3600 mol/m^3^, corresponding to approximately 14% LiCl. Higher concentrations affect permselectivity in the cation exchange membrane, increasing undesired co-ion transport and reducing LiOH product purity.

Regarding process design, it has been found that for the batch process, a low $V_{LiOH}/V_{HCl}$ ratio allows for control of concentration variation in LiOH solutions, thereby contributing to the reduction of Cl^−^ ion leakage in the bipolar membrane. On the other hand, the number of compartments of the electrodialysis stack influences specific electrical energy consumption (SEC) according to the LiOH concentration reached. It has been determined that from 80 compartments the decrease of SEC is less than 1%.

Work carried out permits obtaining a proper approximation of the actual production range of LiOH by BMED at high concentrations according to ion exchange membranes characteristics, operating conditions and phenomena that affect their performance. It has been determined that the use of improved hypothetical ion exchange membranes with low OH^−^ ion leakage increases production efficiency by about 20%, further improving product solution purity. The performed parametric analysis provides key information on process sensitivity to operating conditions, stack and process design. This information can be used for experimental research at the pilot scale.

## Figures and Tables

**Table 1:** Membranes and electrode compartments characteristics used in simulation.

Cation Exchange Membrane		
Ion exchange capacity (meq/gr)	IEC	1.6
Water content	$w_{u}$	29.7–33.5%
Thickness (mm)	Δx	18
Apparent Li^+^ diffusion coefficient (10^−12^ m^2^/s)	$D_{Li}^{cm}$	9.0
Apparent OH^−^ diffusion coefficient (10^−12^ m^2^/s)	$D_{OH}^{cm}$	3–27
Bipolar Membrane		
Fixed charge density anion and cation layer (mol/m^3^)	$C_{fix}^{bpm}$	3500 to 6500
Anion layer thickness (mm)	$\Delta x_{a}$	0.11
Cation layer thickness (mm)	$\Delta x_{c}$	0.11
Diffusion coefficient (10^−12^ × m^2^/s)	$D^{bpl}$	60 to 140
Electrodes Compartments		
Standard electrode potential difference (V)	$\Delta E_{e}$	2.06
Anode overpotential	$\eta_{a}$	0.1
Cathode overpotential	$\eta_{c}$	0.1

**Table 2:** Experimental test setup for validation.

	Configuration 1	Configuration 2	Configuration 3	Configuration 4
Number of compartments	2	2	2	4
Current density	1000 A/m^2^	500 A/m^2^	1000 A/m^2^	500 A/m^2^
Inicial LiCl concentration	3.56 mol/L (aprox 14 %mass)	3.56 mol/L (aprox 14 %mass)	6.77 mol/L (aprox 25 %mass)	9.62 mol/L (aprox 34 %mass)
LiCl volumen solution	450 mL	450 mL	300 mL	450 mL

**Table 3:** BMED stack design parameters and initial operating conditions for parametric study.

Design of Stack		
Number of compartments	N	20
Distance between membranes (m)	d	0.001
Electrode compartment thickness (m)	de	0.02
Effective membrane area (m^2^)	A	0.03
Membrane electric resistance (Ω∙cm^2^)	$R^{mem}$	3.0
Operating conditions		
Initial LiOH concentration (mol/m^3^)	$C_{Li,0}^{LiOH}$	210
Initial LiCl concentration (mol/m^3^)	$C_{Li,0}^{LiCl}$	3600 to 9600
Initial HCl concentration (mol/m^3^)	$C_{Cl,0}^{HCl}$	137
Electrode solution concentration (mol/m^3^)	$C_{0}^{elec\;sol}$	1000
LiOH solution volume (m^3^)	$V_{LiOH}$	0.025
LiCl solution volume (m^3^)	$V_{LiOH}$	0.025
HCl solution volume (m^3^)	$V_{LiOH}$	0.025
Current density (A/m^2^)	i	300 to 2000
Temperature (°C)	T	25

**Table 4:** R^2^ coefficients determination and root-mean-square error between observed and predicted values as per mathematical model.

Configuration	Parameter	$C_{LiOH}$ (%)	$C_{Cl}$ (%)	CE (-)	SEC (kWh/kg)	Purity (%)	Voltage (V)
1N	R^2^	99.883	93.116	99.993	98.147	94.344	97.634
	RMSE	0.30	0.07	0.11	1.82	0.03	0.43
1F	R^2^	99.925	95.407	99.990	99.9999	89.301	94.679
	RMSE	0.11	0.08	0.06	1.04	0.06	0.49
2	R^2^	97.051	99.999	98.974	99.991	98.915	99.407
	RMSE	0.19	0.27	0.01	2.00	0.03	1.29
3X	R^2^	99.331	94.465	99.655	99.289	35.054	67.568
	RMSE	0.43	0.06	0.04	0.89	0.03	0.89
3B	R^2^	99.811	97.248	97.555	96.536	80.743	87.0971
	RMSE	0.23	0.03	0.06	0.57	0.01	1.26
4	R^2^	99.311	97.562	99.857	99.921	99.978	59.405
	RMSE	0.14	0.09	0.07	1.19	0.03	0.40

## Data Availability

Not applicable.

## Nomenclature

J	Ions molar flux
D	Apparent diffusion coefficiente
C	Mole concentration per unit volumen
Δx	Cation exchange membrane thickness
z	Number of electrons per ion
φ	Electric potential
R	Ideal gas constant
T	Temperature
i	Electric current density
F	Faraday Constant
N	Number of compartments
t	time
A	Effective membrane area
λ	Molar conductivity
K	Kohlraush constant
k	Specific conductivity
$R^{s}$	Electric resistance of solution
d	Distance between membranes
de	Electrode compartment thickness
E	Cell potential
η	overpotential
U	Voltage
mp	mass produced
SEC	Specific electrical energy consumption
ϕ	Current efficiency
m	molality
M	Molar mass
V	Solution volume
ρ	density
Subscripts and Superscripts	
cm	Cation membrane
cms	Cation membrane surface
fix	Fixed, associated with fixed charges of the cation exchange membrane or bipolar membrane
i	Ionic or molecular species
s	solution
0	Initial time zero
cou	counterion
co	Co-ion
prod	Production
lim	Related to limiting current density
bpl	Bipolar membrane layer
a	Anionic
c	Cationic
m	molar
°	standar
mem	Membrane
bpm	Bipolar membrane
e	equilibrum
cell	cell
elec sol	Electrolyte solution
